# Evaluation of a randomized controlled trial on the effect on return to work with coaching combined with light therapy and pulsed electromagnetic field therapy for workers with work-related chronic stress

**DOI:** 10.1186/s12889-017-4720-y

**Published:** 2017-10-02

**Authors:** Karen Nieuwenhuijsen, Antonius M.C. Schoutens, Monique H.W. Frings-Dresen, Judith K. Sluiter

**Affiliations:** 1 Department: Coronel Institute of Occupational Health, Academic Medical Center, University of Amsterdam, Amsterdam Public Health research institute, Amsterdam, the Netherlands; 2 FluxPlus BV, Saal van Zwanenbergweg 11, 5026 RM Tilburg, the Netherlands

**Keywords:** Work-related chronic stress, Burnout, Light therapy, PEMF, Return to work, Fatigue, Stress, Quality of life

## Abstract

**Background:**

Chronic work-related stress is quite prevalent in the working population and is in some cases accompanied by long-term sick leave. These stress complaints highly impact employees and are costly due to lost productivity and medical expenses. A new treatment platform with light therapy plus Pulsed Electro Magnetic Fields (PEMF) in combination with coaching was used to assess whether more positive effects on return to work, stress, work-related fatigue, and quality of life could be induced compared to coaching alone.

**Methods:**

A placebo-controlled trial was executed after inclusion of 96 workers, aged 18–65 with work-related chronic stress complaints and who were on sick leave (either part-time or full-time). Participants were divided into three arms at random. Group 1 (*n* = 28) received the treatment and coaching (Intervention group), group 2 (*n* = 28) received the treatment with the device turned off and coaching (Placebo group) and group 3 (*n* = 28) received coaching only (Control group). The data were collected at baseline, and after 6, 12 and 24 weeks. The primary outcome was % return to work, and secondary outcomes were work-related fatigue (emotional exhaustion and need for recovery after work), stress (distress and hair cortisol), and quality of life (SF-36 dimensions: vitality, emotional role limitation, and social functioning).

**Results:**

Eighty-four workers completed all measurements, 28 in each group. All groups improved significantly over time in the level of return to work, as well as on all secondary outcomes. No statistical differences between the three groups were found either on the primary outcome or on any of the secondary outcomes.

**Conclusions:**

Light therapy with Pulsed Electro Magnetic Fields PEMF therapy has no additional effect on return to work, stress, fatigue, and quality of live compared to coaching alone.

**Trial registration:**

NTR4794, registration date: 18-sep-2014

## Background

Work-related chronic stress (burnout) can present itself through the feeling of being very fatigued, accompanied by decreased drive or motivation for carrying out activities related to work. This can lead to problems with functioning [1]. Workers with burnout complaints are known to have little energy to start new undertakings, feel very tired and are no longer able to carry out their daily work routines. [2]. Work-related chronic stress is a growing problem for employees and employers and the costs to society are significant [3]. One of the drivers of these societal costs is sick leave. A Danish study showed that 19% of a cohort of workers on sick leave due to stress and burnout complaints did not return to work within 40 weeks of sick leave [4].

Various professionals can be involved in the treatment of work-related chronic stress problems in the Netherlands, such as psychologists, occupational physicians, general physicians, re-integration counsellors, and coaches. The guidance advised by these professionals for employees with these problems and associated sickness absence is mental coaching, based on cognitive-behavioural and problem-solving understandings [5]. The common goal of guidance through coaching in these workers is to enhance the return to work process. Nevertheless, work-related chronic stress problems are still associated with long absences from work. Roelen and colleagues [6] found a median duration of 109 days in this group. New innovative treatments may be needed to further enhance return to work in workers with chronic stress problems.

In people with burnout, light therapy showed a trend in reducing the symptoms more compared to a waiting list group [7]. However, this evidence stems from a rather small-scale study. In people with mood disorder [8] and pain [9] weak magnetic fields like Pulsed Electromagnetic Fields (PEMFs) have been deployed with varying levels of success in treatments. The combination of light therapy and electromagnetic field therapy in the treatment unit Xentix [10] might be seen as a supplementary treatment. Light therapy is known to alleviate the sleep/wake rhythm, and this may result in more vitality and less exhaustion. Pulsed Electromagnetic Field therapy helps to stabilize the metabolism, resulting in a better balance between the cell and the intercellular spaces [11]. The mechanism of this therapy is thought to be that: i. hormones and neurotransmitters transmit chemical communication from one cell type to another that moderate the metabolic reactions of tissues to the environmental surroundings; ii. communication between these signalling structures is a potential mechanism by which very low-energy electromagnetic fields might increase metabolic activity in the human body. Hormone and neurotransmitter receptors are specific protein molecules that use a diversity of biochemical activities to pass chemical indicators from the external space of a cell through the plasma membrane to the inner space of the cell; iii. since many low-energy electromagnetic fields have less energy to directly pass through the membrane, it is likely that they may modify the existing signal transduction routes in cell membranes, thus producing both transduction and biochemical extension of the properties of the field itself [12]. However, the present understanding of what the physiological mechanisms of PEMF might be is incomplete. As magnetic field therapy in patients with fibromyalgia [13] showed a positive effect on the level of emotional fatigue and light therapy has also been found to have a positive effect on tiredness and quality of life in patients with winter depression (SAD: Seasonal Affective Disorder) and the less severe form, winter blues (Subsyndromal Seasonal Affective Disorder S-SAD) [14, 15], we hypothesised that the combination therapy could help reduce complaints in workers with chronic stress-related problems. In addition, because in Multiple Sclerosis (MS) patients experience a positive effect of light therapy on tiredness [16, 17] and because magnetic field therapy showed a positive influence on quality of life [18], we believed that combining these therapies might also positively influence the return to work process. However, a prerequisite for considering the use of light and magnetic therapy to enhance return to work is that the effect of these treatments would exceed the effects of coaching alone. We therefore hypothesised that an approach incorporating coaching [19, 20] combined with light therapy and pulsed electromagnetic field therapy in people with work-related chronic stress symptoms contributes to an earlier return to work compared to guidance with coaching only. Additionally, we studied whether the intervention with coaching and light therapy plus magnetic field therapy would lead to a greater reduction in stress levels and fatigue and to an improvement of quality of life compared to coaching only.

## Method

### Design

This study used a randomized placebo-controlled trial (RCT) design with three arms in subjects with work-related chronic stress. The study design was described by Schoutens et al. [21]. Participants were randomly allocated to one of three groups: group 1 received light therapy/electromagnetic field therapy and coaching (Intervention group); group 2 received the same treatment conditions but the light therapy/electromagnetic field therapy was not activated (Placebo group); and Group 3 received coaching only (Control group).

### Participants

The total number of participants needed to be able to show an effect size of 0.3 with a power of 0.8 was *N* = 79. This number was based on a sample size calculation of the F-test of a MANOVA with repeated measures (critical F = 2.16). Participants were recruited through social media, newspapers, general practitioners and through an occupational health service. To allow for loss to follow-up, we included a total number of 96 employees (30 males/66 females, between 18 and 65 years of age) from a southern region of the Netherlands. At baseline, socio-demographic data such as age, gender and job title were collected by the research assistants by means of an interview. Participants had to experience work-related chronic stress complaints and be absent from work for at least 50% of their working contract hours. They had to be diagnosed with neurasthenia (symptoms 0–6 months) and able to speak Dutch. Exclusion criteria were pregnancy, serious somatic problems such as diabetes or epilepsy, serious ocular diseases, confusion, or severe depressed mood, use of psychotropic drugs other than selective serotonin reuptake inhibitors SSRIs and pacemaker/neuro-stimulators. In case of a referral by a general or occupational health professional, these inclusion criteria were checked by the professional. In self-referred patients, eligibility was checked by self-report questions. Subjects who met the inclusion criteria were asked to participate in the study and to provide written informed consent. The primary check of the inclusion criteria was conducted by the research assistant. For all included participants, this was checked by the coach in an intake meeting.

### Setting

**Fig. 1:**
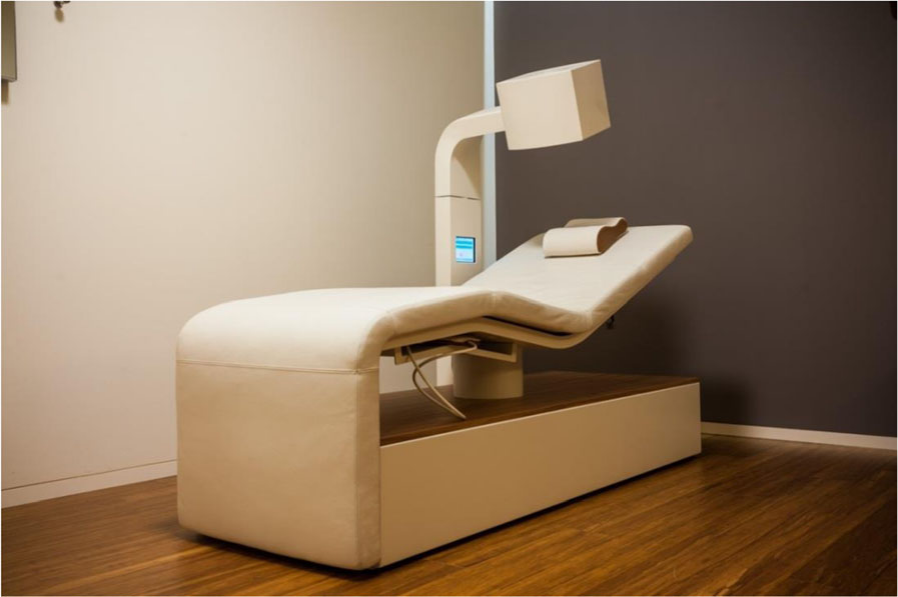
Xentix treatment platform

The study was conducted in a treatment centre in the southern part of the Netherlands. This centre was built and equipped specifically for this study. The centre offered five treatment rooms where the treatment equipment was set up, and two rooms for coaching sessions. The device used in the study was a reclining chair with an extended seat forming a leg rest (see Fig. 1). It formed a comfortable treatment platform with a light therapy unit mounted above the participant’s head. The electromagnetic coils were situated in the pillow and bed.

### Procedure

The procedure is described below as was done in the design study of Schoutens et al. [21]. Randomization took place as soon as the participant met the inclusion criteria, gave written informed consent, and filled in the baseline measurements. Preliminary numbers were assigned to the three groups of equal size in the study. Participants random received a number from an assistant who was not involved in the actual measurements. All numbers were written on the measuring instruments. With this number, the participants were allocated to one of the three groups. During the treatment, the participants from the Intervention and Placebo groups lay down on the bed of the treatment platform. Their eyes were closed so that the light reached the retina through their eyelids. In the case of high photosensitivity of the participant, dark goggles could be worn. Participants came to the research centre and were treated for 12 weeks, twice a week for 40 min on the treatment platform with light therapy/magnetic field therapy (Intervention group). The Placebo group received the same treatment condition but the light and magnetic field were switched off (Placebo group). Only a non-effective, small dose of coherent/incoherent light >5 lx was used to give the impression that the equipment was running. The Control group did not lie down on the bed but received coaching only (Control group), also for 50 min once a fortnight during a 12-week period. The coaching that all participants received was regular guidance during coaching sessions, provided by a certified coach. Coaching was provided for 50 min every fortnight during a 12-week period.

### Measurements

#### Primary outcome

##### 2.5.1.1.Return to work

The primary outcome was the percentage return to work: the number of their worked hours per week at the end of the study compared to the number of contract hours they had in the week prior to the study (T0). Return to work was assessed at T0 (start), T2 (after 12 weeks) and T3 (after 24 weeks). Scores ranged between 0 (no working hours) and 100 (the number of contract hours working).

#### Secondary outcomes

**Fatigue** was assessed using two scales. The first scale assessed *emotional exhaustion*, a scale with five items that is part of the Dutch UBOS G(eneral) [22]. The Utrecht Burnout Scale UBOS is the Dutch version of the Maslach Burnout Inventory. The scale score ranged between zero and six, with higher scores indicating more emotional exhaustion.

The *Need for Recovery* after work scale of the Dutch Experience and Evaluation of Work Questionnaire (Dutch: VBBA, Meijman and Van Veldhoven, 1994) [23] assessed work-related fatigue: the extent to which employees experience problems in recovery efforts from work. The scale score was calculated by adding the individual’s scores on the 11 items and transforming this score into a scale score ranging from 0 (no need for recovery) to 100 (maximum need for recovery).

**Stress** was measured using two instruments: first, the *distress scale* of the Four-Dimensional Symptoms Questionnaire (4DSQ) [24] was used. The 16-item questionnaire uses a 5-point response scale (0 = *no*, 4 = *very often*). Second, the accumulation of the stress level of an individual was assessed by the *stress hormone cortisol in hair*. Hair cortisol reflects the cumulative responses over time of cortisol in the body [25]. Hair strands in the posterior vertex region were cut as close as possible to the scalp using iron scissors. Cortisol concentrations were determined from two 1-cm strands from a 3-cm hair segment that was cut. Based on an average hair growth rate of 1 cm per month [26], we used two hair segments to reflect the cortisol concentration at baseline (T0) and the cortisol concentration in the month before T2.

**Quality of life** was measured using the Dutch version of the SF-36 questionnaire [27]. We used three dimensions from this questionnaire: vitality, emotional role limitations, and social functioning. For each dimension, the scale score was calculated, with scores between 0 (worst score) and 100 (best score).

### Intervention

The principle of operation of the treatment platform was, according to the manufacturer, as follows: the light therapy unit consisted of a combination of coherent and incoherent light. The coherent light was generated through multiple high-performance light emitting diodes (LEDs) with wavelengths of 470 nm (blue), 525 nm (green) and 570 nm LED (yellow). The non-coherent light was generated by means of an optically-centred halogen lamp with a light output of +/− 9000 lm. The maximum illuminance at the eye was +/− 6000 lx. The light was directed by means of facetted and coated reflectors and then linearly polarized. Ultra Violet (UV) and Infra-Red (IR) wavelengths were filtered out of the spectrum.

The weak magnetic fields were generated in the device using low voltage in multiple coils integrated and spread over the sofa and in the pillow set. The magnetic fields are wide-bands and the amplitude of the magnetic fields is almost constant across the used frequency band. The specific frequency band is in the ELF (extremely low frequency, 0-30 Hz) and the ULF (ultra-low frequency, 30–30,000 Hz) range. The maximum magnetic flux density B = 0,3 Wb/m^2^ = 0,3 T.

Coaching was performed using a standard guidance protocol with person-directed interventions to reduce burnout and to improve occupational mental health. The guidance helps in identifying and solving the mental problems the employee has. During coaching, three stages of recovery were defined and followed: acceptation and relaxation, employability, and support to return to work. Intervention elements include cognitive skills training and examining dysfunctional thoughts which perpetuate the symptoms. In addition, psycho-education, organisation-oriented interventions, conflict- and time management and therapies that influence fatigue and stress were deployed. Coaching was provided by qualified professionals.

### Statistical analysis

Descriptive statistics were presented as percentage, mean, standard deviation (SD) and range for each group separately. Baseline differences between groups were tested with independent sample T-tests for normally distributed and Kruskal-Willis for non-normally distributed parameters. Baseline differences in percentages were tested with a Chi-Square test. To test for differences between the study arms over time, for each outcome an analysis of variance (GLM repeated measures) was planned. Each outcome and their residuals when entered in GLM was checked for normality. In the case of a clear skewed distribution, we used a log transformation to normalize the data. In cases where log transformations did not suffice, the effects were tested using non-parametric tests. Friedman test was then used to test differences over time for the group as a whole and the Kruskal-Wallis test was used to compare the outcomes of the groups at each of the time points. All analyses were conducted using the statistical package IBM SPSS Statistics 23.

### Ethics, consent and permissions

The study has been approved by the Medical Ethics Assessment Committee of the Academic Medical Center (University of Amsterdam; Ref. METC 2014_260 #B2014922). Written informed consent was provided by all participants prior to enrolment. There are no risks associated with participating in the study due to the light intensity being lower than daylight and not including Ultra Violet or Infra Red wavelengths and the use of weak electromagnetic fields. No restrictions to other care of usual were in place for any study participant.

## Results

A total of 84 out of the 96 included participants stayed in the study and performed all measurements, 28 in all three groups. In Table 1, the demographics of these 84 participants are shown, per group. The checks of the research assistant and the coach revealed that none of the participants had any physical or psychiatric disorders, for which psychotropic medication other than serotonin reuptake inhibitors was needed. The mean age of the participants was 43 (SD 8), 47 (SD 9.7), and 40 (8.9) in the intervention, placebo, and control group respectively.. About two-thirds of the participants were females and participants worked between 0% to 63% of their contract hours (median 0%).. The three groups did not differ in the proportion of males and females in each group nor did they differ in the percentage of working hours at baseline. However, age was significantly different across the three groups (*p* = 0.01).

**Table 1:** Demographics: age, gender and percentage of working hours at work at baseline, per group

		Mean(sd)	Percentage (%)	Median (IQR)	*p*-value of between-groups test*
Age (years)	Intervention (*n* = 28)	43 (8.0)			0.01
	Placebo (*n* = 28)	47 (9.7)			
	Control (*n* = 28)	40 (8.9)			
Gender (% women)	Intervention (*n* = 28)		72		0.87
	Placebo (*n* = 28)		69		
	Control (*n* = 28)		66		
Work-hours RTW at baseline (% of contract hours)	Intervention (*n* = 28)	7.3 (16.70)		0.0 (1.25)	0.17
	Placebo (*n* = 28)	13.2 (20.44)		0.0 (24.31)	
	Control (*n* = 28)	6.9 (17.80)		0.0 (0.00)	

*Independent sample T-test for age, Chi-square test for gender and Kruskal-Wallis for Work-hours RTW

The primary outcome return to work, and the secondary outcome Need for Recovery after work had to be analysed non-parametrically as these outcomes showed a highly skewed distribution. Log transformations did not yield normally distributed outcomes. We therefore used a Kruskal-Wallis to test for group differences at each timepoint and a Friedman test for differences over time for all groups combined. The secondary outcome hair cortisol could be analysed with GLM after a log transformation. For the secondary outcome measures of emotional exhaustion, distress and quality of life, no transformations were needed.

In Table 1, the descriptives at baseline are shown for all outcomes per group. Table 2 presents the scores of primary and secondary outcomes in three groups over time. For the primary outcome, it shows that the percentage of working hours increased in all groups up to about two-thirds of their contract hours on average. This main effect over time was statistically significant. For the secondary outcomes, we found that emotional exhaustion complaints and stress complaints were on average substantially and significantly decreased after treatment compared to the start, in all groups (main effect over time). The trends in cortisol concentrations were not equal in all groups: the average cortisol level decreased in the Placebo group but increased in both the Intervention group and in the Control group. Work-related fatigue levels did decrease significantly after treatment in all groups – to about half the level it was when the study started. For quality of life, we found a similar effect over time – vitality levels increased as did social functioning, and emotional role limitations decreased, also in all groups and significantly.

**Table 2:** Median (IQR) or mean (sd) scores of primary and secondary outcomes in three groups over time

Outcomes:		Baseline	Week 6	Week 12 (after treatment)	Week 24	*p*-value between groups over time***	*p*-value within groups over time
Primary outcome: Median (IQR)							
% work-hours RTW (0–100)**	Intervention	0.0 (1.25)	–	22.5 (66.67)	94.7 (80.63)	0.92	
	Placebo	0.0 (24.31)	–	14.3 (64.50)	88.2 (58.54)		
	Control	0.0 (0.00)	–	25.0 (52.50)	62.5 (72.31)		
	Total group	0.0 (4.06)		20.0 (60.56)	87.5 (70.94)		0.00
Secondary outcomes:							
Emotional exhaustion*	Intervention	4.9 (1.28)	–	3.2 (1.46)		0.20	
	Placebo	5.2 (1.26)	–	2.6 (1.30)			
	Control	5.3 (0.89)	–	3.2 (1.63)			
	Total group	5.1 (1.16)		3.0 (1.47)			0.00
Work-related fatigue(0–100)*Median (IQR)	Intervention	90.9 (27.27)	72.7 (50.0)	40.9 (59.09)	–	0.70	
	Placebo	90.9 (15.91)	72.7 (43.18)	40.9 (52.27)	–		
	Control	90.9 (9.09)	81.8 (50.0)	50.0 (68.18)	–		
	Total group	90.9 (25.0)	81.8 (43.18)	45.5 (63.6)			0.00
Vitality (QoL) (0–100)**	Intervention	31.7 (18.82)	44.3(18.60)	52.7 (21.32)	–	0.20	
	Placebo	29.2 (17.80)	46.6(17.95)	63.2 (16.40)	–		
	Control	27.8 (13.50)	40.5(16.26)	52.1(18.88)	–		
	Total group	29.6 (16.76)	43.7 (17.59)	53.0 (19.43)			0.00
Stress (0–64)*	Intervention	22.0 (7.47)	16.3(7.43)	12.0 (7.36)	–	0.95	
	Placebo	21.9 (8.34)	14.1(8.20)	9.4 (7.41)	–		
	Control	23.0 (8.01)	16.6(9.51)	13.1 (9.29)	–		
	Total group	22.3 (7.88)	15.7 (8.40)	11.5 (8.12)			0.00
Cortisol (pg/mg)	Intervention	32.2 (38.48)	–	39.1 (44.38)	–	0.40	
	Placebo	38.8 (85.58)	–	30.1 (26.71)	–		
	Control	20.5 (15.44)	–	30.0 (19.36)	–		
	Total group	29.9 (52.60)		32.0 (31.09)			0.00
Emotional role limitation (QoL) (0–100)**	Intervention	21.1 (28.34)	35.6 (37.72)	64.3 (32.62)	–	0.96	
	Placebo	22.6 (27.74)	46.4 (39.90)	67.9 (33.31)	–		
	Control	17.8 (17.78)	30.0 (36.46)	58.3 (39.15)	–		
	Total group	20.5 (28.00)	37.2 (38.18)	63.5 (34.95)			0.00
Social Functioning (QoL) (0–100)**	Intervention	44.6 (30.39)	55.6 (18.78)	68.8 (22.69)	–	0.71	
	Placebo	44.4 (23.90)	62.1 (24.17)	74.1 (20.67)	–		
	Control	41.7 (22.10)	49.2 (23.20)	66.5 (23.20)	–		
	Total group	43.5 (25.44)	55.5 (22.53)	69.8 (22.40)			0.00

*higher scores reflect worse outcomes

**higher scores reflect better outcomes

***For non-parametric tests, only p-value of the between group difference at the last data point is presented

In Table 2 it is also shown that our analysis of between-groups effects over time did not confirm our hypothesis that an approach incorporating coaching combined with light therapy plus pulsed electromagnetic field therapy in people with work-related chronic stress symptoms contributes to an earlier return to work compared to guidance with coaching only. No significant between-groups effect over time was found (*p* = 0.71). Also, our secondary hypothesis could not be confirmed because we did not find that the intervention with coaching and light therapy plus magnetic field therapy led to more reduction in stress levels and fatigue or to an improvement of quality of life compared to coaching only: no significant between-groups effects were found for fatigue, or for stress or quality of life.

## Discussion

Contrary to our expectations, this study did not show a positive effect of combining mental coaching with light therapy plus pulsed electromagnetic field therapy compared to mental coaching alone (with or without placebo treatment) on return to work. On average, workers had returned to two-thirds of their contract hours. We also did not find that the combination of coaching and light plus electromagnetic therapy had positive effects on any of the secondary outcomes of emotional exhaustion, work-related fatigue, vitality, stress, hair cortisol, emotional role limitations and social functioning. These secondary outcomes all show comparable improvements over time for all three groups, with the exception of hair cortisol, where the Placebo group seemed to change in the opposite direction of the other two comparison groups, although these changes were not significant.

A strength of the study was that despite its rigorous RCT study design, the lack of strict diagnostic inclusion criteria allowed for inclusion of the range of patients with work-related chronic stress, contributing to the external validity of the results. Another feature contributing to the external validity of our findings was the decision to include comparison groups with coaching rather than a no-treatment Control group. For the light and magnetic field treatment to be useful in practice, its results should exceed the results of usual care, which in this case includes coaching.

While the results do not point to a positive effect of the tested combination of light therapy plus Pulsed Electromagnetic Field therapy, the implications of our findings are not straightforward. One reason for this is that the treatment platform encompassed two working mechanisms – light therapy and pulsed electromagnetic field therapy. While we were unable to replicate the positive findings of the two studies using only one of these elements [7, 8], it is not clear whether these two mechanisms may have negatively influenced each other. Moreover, in hindsight, the light intensity may have been insufficient and it is unclear whether the instructions using this platform to the participants to close their eyes during the intervention might have counteracted possible effects. As a result, the light may not have been sufficiently able to reach the expected areas in the brain. Further, as the use of electromagnetic fields in the treatment of mental health problems is newly emerging, knowledge on the threshold for a therapeutic effect is actually still lacking. In addition, the choice in the use of safe but very low frequencies may not have been sufficiently high to ensure therapeutic effects in workers with chronic stress complaints.

## Conclusion

Mental coaching with light therapy plus pulsed electromagnetic field therapy did not have a positive effect on any of the outcomes, compared to mental coaching with or without a placebo treatment. It can be concluded that the light plus electromagnetic field therapy is advised not to be implemented in (occupational) health care in its current form. Future proof of concept studies should be conducted with various settings for the magnetic field therapy and with eyes open instructions for the light therapy before testing their effects in further randomized controlled trials.

## Abbreviations

klx: Kilolux
lx: Lux
PEMF: Pulsed Electromagnetic Fields
RCT: Randomized Controlled Trial
SAD: Seasonal Affective Disorder
S-SASD: Subsyndromal Seasonal Affective Disorder
UBOS: Utrecht Burnout Scale

## References

[1] Maslach C, Schaufeli WB, Leiter MP (2001). Job burnout. Annu Rev Psychol.

[2] Sluiter JK, de Groene G, Nieuwenhuijsen K (2013). Burnout als beroepsziekte [Burnout as an occupational disease]. Tijdschrift voor Arbeidsvraagstukken.

[3] Schaufeli WB, Bakker AB (2007). De psychologie van arbeid en gezondheid [the psychology of work and health].

[4] Nielsen MB, Madsen IE, Bultmann U, Christensen U, Diderichsen F, Rugulies R (2011). Predictors of return to work in employees sick-listed with mental health problems: findings from a longitudinal study. Eur J Pub Health.

[5] Bastiaanssen MLM, Terluin B, Vendrig L, Verschuren C, Vrieze J (2011). National primary care guideline neurasthenia and burn-out [*In dutch: Landelijke Eerstelijns Samenwerkings Afspraak Overspanning en burn-out*]. Huisarts & Wetenschap.

[6] Roelen CA, Norder G, Koopmans PC, van Rhenen W, van der Klink JJ, Bultmann U (2012). Employees sick-listed with mental disorders: who returns to work and when?. J Occup Rehabil.

[7] Meesters Y, Waslander M (2009). Burnout and light treatment. Stress Health.

[8] Martiny K, Lunde M, Bech P (2010). Transcranial low voltage pulsed electromagnetic fields in patients with treatment-resistant depression. Biol Psychiatry.

[9] Kortekaas R, van Nierop LE, Baas VG, Konopka KH, Harbers M (2013). A novel magnetic stimulator increases experimental pain tolerance in healthy volunteers - a double-blind sham-controlled crossover study. PLoS One.

[10] Vancraeyenest M. System for influencing of a biological cellular structure. United States Patent. 2010;7(744):522.

[11] Rahbek UL, Tritsaris K, Dissing S (2013). Interactions of low frequency, pulsed electromagnetic field with living tissue: biochemical responses and clinical results. Oral Biosci Med.

[12] Luben RA (1991). Effects of low-energy electromagnetic fields (pulsed an DC) on membrane signal transduction processes in biological systems. Health Phys.

[13] Sutbeyaz ST, Sezer N, Koseoglu F, Kibar S (2009). Low-frequency pulsed electromagnetic field therapy in fibromyalgia: a randomized, double-blind, sham-controlled clinical study. Clin J Pain.

[14] Golden RN, Gaynes BN, Ekstrom RD, Hamer RM, Jacobsen FM, Suppes T, Wisner KL, Nemeroff CB (2005). The efficacy of light therapy in the treatment of mood disorders: a review and meta-analysis of the evidence. Am J Psychiatry.

[15] Rastad C, Ulfberg J, Lindberg P (2011). Improvement in fatigue, sleepiness, and health-related quality of life with bright light treatment in persons with seasonal affective disorder and Subsyndromal SAD. Depress Res Treat.

[16] Reuven S (1996). Treatment with weak electromagnetic fields improves fatigue associated with multiple sclerosis. Int J Neurosci.

[17] Richards TL, Lappin MS, Acosta-Urquidi J, Kraft GH, Heide AC, Lawrie FW, Merrill TE, Melton GB, Cunningham CA (1997). Double-blind study of pulsing magnetic field effects on multiple sclerosis. J Alternat Complement Med.

[18] Lappin MS, Lawrie FW, Richards TL, Kramer ED (2003). Effects of a pulsed electromagnetic therapy on multiple sclerosis fatigue and quality of life: a double-blind, placebo-controlled trial. Altern Ther Health Med.

[19] Theeboom T, Beersma B, van Vianen AEM (2014). Does coaching work? A meta-analysis on the effects of coaching on individual level outcomes in an organizational context. J Posit Psychol.

[20] Jones RB, Woods SA, Guillaume YRF (2015). The effectiveness of workplace coaching: a meta-analysis of learning and performance outcomes from coaching. J Occup Organ Psychol.

[21] Schoutens AMC, Frings-Dresen MHW, Sluiter JK (2016). Design of a randomized controlled trial on the effect on return to work with coaching plus light therapy and pulsed electromagnetic field therapy for workers with work-related chronic stress. BMC Public Health.

[22] Schaufeli WB, van Dierendonck D (2000). UBOS Utrechtse Burnout Schaal Handleiding [Utrecht Burnout Scale Manual].

[23] Van Veldhoven MJPM, Meijman TF: Het meten van psychosociale arbeidsbelasting met een vragenlijst: de Vragenlijst Beleving en Beoordeling van de Arbeid (VBBA) [The measurement of psychosocial job demands with a questionnaire: the questionnaire on the experience and evaluation of work (QEEW)] Amsterdam: Dutch Institute for Working Conditions; 1994.

[24] Terluin B, van Marwijk HWJ, Adèr HJ, de Vet HCW, Penninx BWJH, Hermens MLM, van Boeijen CA, van Balkom AJLM, van der Klink JL, Stalman WAB (2006). The Four-Dimensional Symptom Questionnaire (4DSQ). A questionnaire to measure distress, depression, anxiety, and somatization. BMC Psychiatry.

[25] Gow R, Thomson S, Rieder M, Van Uum S, Kore G (2010). An assessment of cortisol analysis in hair and its clinical applications. Forensic Sci Int.

[26] Stalder T, Kirschbaum C (2012). Analysis of cortisol in hair – state of the art and future directions. Brain Behav Immun.

[27] Ware JE (1998). Overview of the SF-36 health survey and the International Quality of Life Assessment (IQOLA) project. J Clin Epidemiol.

